# Comparative enthesitis burden in psoriatic arthritis vs axial spondyloarthritis: a cross-sectional analysis of the Swiss Clinical Quality Management in Rheumatic Diseases (SCQM) registry

**DOI:** 10.1016/j.ero.2026.100218

**Published:** 2026-07-19

**Authors:** Felix Doll, Adrian Ciurea, Sabine Adler, Laure Brulhart, Frauke Förger, Thomas Hügle, Diego Kyburz, Burkhard Möller, Michael J. Nissen, Giorgio Tamborrini, Oliver Distler, Julia-Tatjana Maul, Raphael Micheroli

**Affiliations:** 1 Department of Rheumatology, University Hospital Zurich, University of Zurich, Zurich, Switzerland; 2 Department of Rheumatology and Immunology, Cantonal Hospital Aarau, Aarau, Switzerland; 3 Department of Rheumatology, Neuchâtel Hospital, La Chaux-de-Fonds, Switzerland; 4 Department of Rheumatology, HOCH Health Ostschweiz, Cantonal Hospital of St. Gallen, St. Gallen, Switzerland; 5 Department of Rheumatology, University Hospital Lausanne (CHUV) and University of Lausanne, Lausanne, Switzerland; 6 Department of Rheumatology, University Hospital Basel, Basel, Switzerland; 7 Department of Rheumatology and Immunology, Bern University Hospital, University of Bern, Bern, Switzerland; 8 Department of Rheumatology, Geneva University Hospital, Geneva, Switzerland; 9 UZR, Swiss Ultrasound Center and Institute for Rheumatology, Basel, Switzerland; 10 Department of Dermatology and Venereology, University Hospital Zurich, University of Zurich, Switzerland

## Abstract

**Objectives:**

This study aimed to compare prevalence, anatomical distribution, and associated factors of enthesitis in psoriatic arthritis (PsA) and axial spondyloarthritis (axSpA) in routine care.

**Methods:**

Cross-sectional analysis of the Swiss Clinical Quality Management in Rheumatic Diseases (SCQM) registry. Patients with PsA or axSpA enrolled from 2018 to 2024 with an available Maastricht Ankylosing Spondylitis Enthesitis Score (MASES) recorded at first visit during study period were included. Enthesitis was defined as a MASES score of > 0. Axial PsA (axPsA) was analysed descriptively for anatomical distribution. Multivariable negative binomial regression modelled entheseal burden, adjusting for age, sex, body mass index (BMI), C-reactive protein levels, symptom duration, and current biologic or targeted synthetic disease-modifying antirheumatic drug use.

**Results:**

We included 3885 patients (PsA, 1473; and axSpA, 2412). Enthesitis prevalence was 56.6% in PsA and 64.9% in axSpA. Among patients with any enthesitis, mean MASES score was 2.8 in PsA, 3.0 in axPsA, and 3.2 in axSpA. Posterior superior iliac spine and L5 spinous process were the most frequently affected sites. Achilles tendon involvement was more prominent in PsA and axPsA, whereas costochondral sites were more frequent in axSpA. Female sex, elevated C-reactive protein levels, higher BMI levels, and current smoking were consistently associated with higher enthesitis burden. Longer symptom duration and biologic disease-modifying antirheumatic drug use were associated with lower burden. No differential effects of BMI or smoking by diagnosis or sex were detected.

**Conclusions:**

Enthesitis is common in PsA and axSpA, with distinct anatomical patterns but largely shared associated factors. Obesity, smoking, and systemic inflammation may represent relevant targets for intervention alongside pharmacologic management, though longitudinal studies are needed to confirm these associations.

**WHAT IS ALREADY KNOWN ON THIS TOPIC**

Enthesitis is a hallmark feature across the spondyloarthritis spectrum, but most evidence on its prevalence, anatomical pattern, and associated factors comes from clinical trials or small cohorts rather than routine care, and direct comparisons between psoriatic arthritis and axial spondyloarthritis remain limited.

**WHAT THIS STUDY ADDS**

In a large real-world registry, enthesitis was common in both diseases, with axial spondyloarthritis showing a greater burden at axial sites and psoriatic arthritis and axial psoriatic arthritis showing more Achilles tendon involvement; female sex, elevated CRP, higher BMI, and current smoking were associated with higher enthesitis burden in both diseases, with no differential effect of BMI or smoking by diagnosis or sex.

**HOW THIS STUDY MIGHT AFFECT RESEARCH, PRACTICE OR POLICY**

These findings support incorporating a full entheseal assessment into routine care for psoriatic arthritis and axial spondyloarthritis and highlight obesity and smoking as potential modifiable targets for intervention, while indicating a need for longitudinal studies to clarify causality.

Alt-text: Unlabelled box dummy alt text

## INTRODUCTION

Enthesitis, defined as inflammation at the insertion sites of tendons, ligaments, and joint capsules at the axial and peripheral skeleton, is a unifying hallmark of the spondyloarthritis (SpA) spectrum and contributes significantly to disease burden [1]. SpA encompasses a group of chronic inflammatory rheumatic diseases, most prominently axial spondyloarthritis (axSpA) and psoriatic arthritis (PsA), which present with heterogeneous clinical manifestations [2]. In addition to enthesitis, SpA is characterised by articular involvement and a broad spectrum of extra-articular manifestations, including dactylitis, uveitis, inflammatory bowel disease, and psoriatic skin and nail involvement [3]. Axial involvement in PsA, commonly referred to as axial PsA (axPsA), represents a heterogeneous and clinically relevant disease phenotype that differs from both peripheral PsA and axial SpA [4,5].

From a clinical perspective, enthesitis is associated with higher overall pain levels, functional impairment, fatigue, and reduced quality of life [6,7]. It may also induce local structural changes at sites of prior inflammation, including bone erosions, calcifications, and new bone formation (enthesiophytes) [8]. Mechanical stress and local immune responses are thought to drive entheseal inflammation. Key mediators and cytokines involved in the inflammatory response at the enthesis, including prostaglandin E_2_, interleukin (IL) 17 (IL-17), IL-23, and tumour necrosis factor (TNF), are responsive to specific therapies, underscoring the relevance of key mediators and cytokines as a clinical therapeutic target [8]. The presence of subclinical entheseal inflammation (eg, detectable using ultrasound or magnetic resonance imaging) in patients with psoriasis suggests that enthesitis may constitute an early manifestation bridging psoriasis and PsA [9]. For clinical assessment, several enthesitis indices are available, including the 3 most common ones, the Spondyloarthritis Research Consortium of Canada (SPARCC) Enthesitis Index, the Leeds enthesitis index, and the Maastricht ankylosing spondylitis enthesitis score (MASES), which differ in number and location of entheseal sites evaluated, with SPARCC providing broader peripheral coverage, the Leeds enthesitis index focusing on peripheral sites, and MASES scores emphasising predominantly axial locations [10]. Reflecting its clinical importance, enthesitis has been included in the classification criteria for SpA, within the Assessment of SpondyloArthritis International Society criteria for axSpA and the Classification Criteria for Psoriatic Arthritis for PsA [11,12].

Despite its clinical relevance, evidence on the prevalence, anatomical distribution, and determinants of enthesitis in axSpA and PsA remains limited. Much of the available literature comes from randomised clinical trials or small observational cohorts and may not reflect routine clinical practice. In particular, the impact of demographic and clinical characteristics and modifiable lifestyle factors (eg, smoking or obesity) on entheseal burden—and whether these associations differ between PsA and axSpA—remains unclear. The Swiss Clinical Quality Management in Rheumatic Diseases (SCQM) registry provides a large real-world dataset to address these gaps and enables robust comparative analyses across disease groups.

The objective of this study was to address current knowledge gaps regarding the burden and distribution of enthesitis in PsA and axSpA. We investigated the prevalence and anatomical distribution of enthesitis and examined demographic, clinical, and disease-related factors linked to entheseal involvement. We also assessed whether the influence of modifiable risk factors, such as body mass index (BMI) and smoking, differs between the 2 diseases. By contrasting enthesitis profiles in large, well-characterised cohorts, this study aimed to refine disease phenotyping, facilitate earlier identification of high-risk patients, and inform personalised management and future research.

## METHODS

### Data source

The SCQM registry in rheumatic diseases is a nationwide longitudinal registry initiated in 2005 for axSpA and 2006 for PsA that prospectively collects routine-care data on inflammatory rheumatic diseases in Switzerland. At each visit, rheumatologists record demographics, clinical assessments, laboratory markers, and treatment exposures, complemented by standardised patient-reported outcomes measures (PROMs). All participants provide written informed consent for the use of pseudonymised data for research and may withdraw at any time.

### Study population

This cross-sectional comparative study used SCQM data from patients with a rheumatologist diagnosis of axSpA or PsA. We included visits recorded between January 1, 2018 and December 31, 2024. The index visit was defined as the first visit in this period with a complete MASES score assessment. No exclusions were applied based on treatment, comorbidities, or fibromyalgia. For anatomical distribution analyses, patients with PsA with physician-reported axial involvement were classified as axPsA. For all other analyses, patients with axPsA were included in the PsA cohort. Regression analyses were restricted to index visits with complete data for prespecified confounders (age, sex, BMI, C-reactive protein [CRP] levels, symptom duration, and current exposure to biologic or targeted synthetic (b/ts) disease-modifying antirheumatic drugs [DMARDs]).

### Patient and public involvement

A patient research partner (Loris Bornacin) contributed to the study design, interpretation of results, and reporting.

### Patient characteristics, clinical assessments, patient-reported outcomes, and enthesitis examination

At the index visit, we extracted demographic characteristics, clinical assessments, laboratory marker levels, treatments, and standardised PROMs. Variables included age, sex, symptom duration, smoking status, human leukocyte antigen B27 (HLA-B27) status, BMI, CRP levels, erythrocyte sedimentation rate, and current treatment (DMARDs, nonsteroidal anti-inflammatory drugs [NSAIDs], and cardiovascular medication). Disease activity measures included tender and swollen joint counts, physician global assessment and ankylosing spondylitis disease activity score-CRP level (axSpA), alongside PROMs (Bath ankylosing spondylitis disease activity index, Bath ankylosing spondylitis functional index, health assessment questionnaire, and patient global assessment). Disease manifestations, including peripheral arthritis, dactylitis, uveitis, psoriatic skin, and nail manifestations, were assessed by the treating rheumatologist. The MASES score comprises 13 sites (6 bilateral sites plus the fifth lumbar spinous process). Clinicians recorded tenderness at each MASES score site in the SCQM registry online database; plantar fascia tenderness was recorded separately and was not included in the primary definition of enthesitis.

### Statistical methods

Patients were analysed by diagnosis (PsA and axSpA) and enthesitis status. Enthesitis was defined as a MASES score > 0; plantar fascia tenderness was analysed descriptively and excluded from the primary definition. Categorical variables are reported as n (%) and continuous variables as mean ± SD. Between-group comparisons used χ² tests or Mann–Whitney U tests, as appropriate. Multivariable negative binomial regression with robust standard errors estimated incidence rate ratios (IRRs) for the number of affected entheseal sites, comparing axSpA with PsA and identifying associated factors. Facilitating clinical interpretation of effect estimates, the expected number of affected sites was reported. Models were adjusted a priori for age, sex, BMI, CRP levels, symptom duration, and current b/tsDMARDs use. Covariates were predefined before the analyses and selected according to their expected relevance for entheseal involvement and their potential to influence the association between disease characteristics and enthesitis burden. HLA-B27 was not included as a covariate because the diagnosis term (PsA vs axSpA) already captures the large majority of HLA-B27-related variance in this population; including both would introduce substantial collinearity. Regression analyses were restricted to patients with complete data for all prespecified covariates (complete case analysis). Effect modification by diagnosis and sex was assessed via interaction terms. Analyses were conducted in R; 2-sided *P* < .05 was considered statistically significant.

As a sensitivity analysis, all descriptive and regression analyses were repeated after excluding patients receiving b/tsDMARDs at the index visit.

### Ethics

This study was approved by the Ethics Committee of the Canton of Zurich (BASEC 2022-00272). All participants provided written informed consent for the use of pseudonymised SCQM registry data for research. The study was conducted in accordance with the Declaration of Helsinki.

## RESULTS

### Patient selection

During our study period, 3885 patients had a MASES score assessment documented at least once. Of these patients, 1473 were classified as having PsA and 2412 as having axSpA. Clinical enthesitis was present in 833 patients with PsA (56.6%) and 1565 patients with axSpA (64.9%), with the corresponding prevalences shown in Figure 1. The model-estimated expected number of affected sites was 1.58 (SE, 0.07) in patients with PsA and 2.23 (SE, 0.07) in patients with axSpA when considering all patients with and without enthesitis. Among patients with enthesitis, the model-estimated expected number was 2.70 (SE, 0.09) for PsA and 3.25 (SE, 0.09) for axSpA, as shown in Figure 1. The number of affected entheseal sites was higher in axSpA than in PsA, both across all patients (IRR, 1.41; 95% CI, 1.26-1.57; *P* < .001) and among patients with enthesitis (IRR, 1.21; 95% CI, 1.10-1.32; *P* < .001).

**Figure 1:**
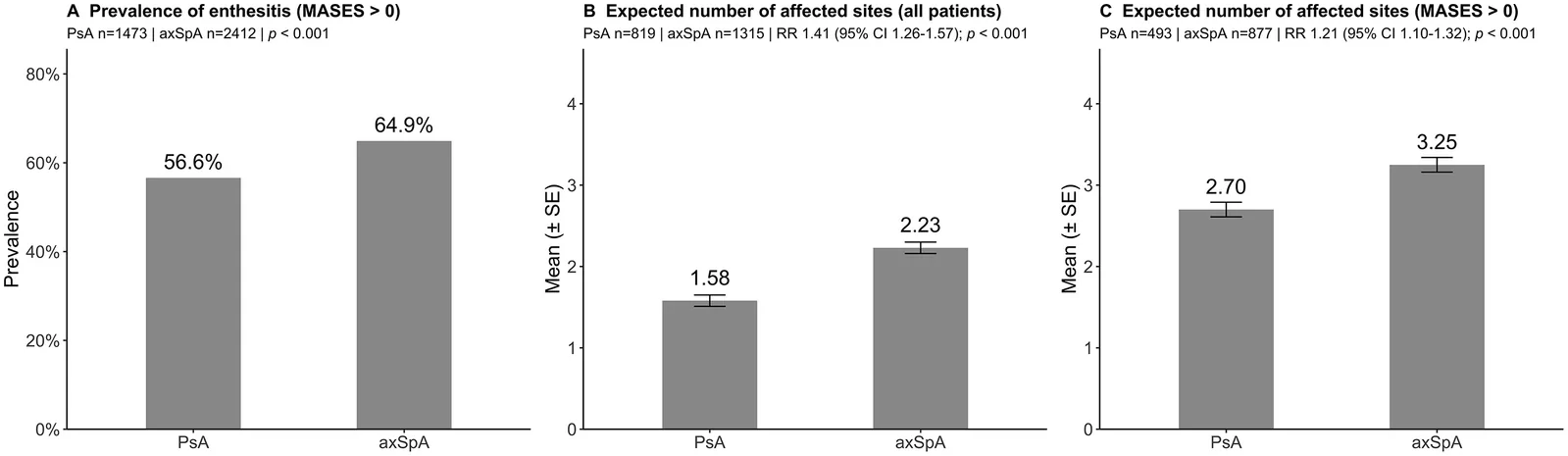
Enthesitis prevalence and model-estimated expected number of affected sites. axSpA, axial spondyloarthritis; IRR, incidence rate ratio; MASES, Maastricht ankylosing spondylitis enthesitis score; PsA, psoriatic arthritis.

### Patient characteristics

Baseline demographic characteristics, clinical characteristics, disease activity, key manifestations, and current treatments at the index visit by diagnosis and enthesitis status are summarised in Table 1. In both PsA and axSpA, patients with enthesitis were more often female and had higher BMI and CRP levels compared with patients without enthesitis. Patients with enthesitis had significantly higher tender joint count (44 joints) and swollen joint count (44 joints), as well as higher physician and patient global assessment scores, compared with patients without enthesitis in both disease groups.

**Table 1:** Baseline characteristics at the index visit by diagnosis and enthesitis status (MASES scores > 0 vs 0)

	PsA		axSpA	
Characteristic	MASES score > 0 (n = 833)	MASES score = 0 (n = 640)	MASES score > 0 (n = 1565)	MASES score = 0 (n = 847)
Demographics and clinical characteristics				
Age, y	53.4 ± 12.6	55.2 ± 12.7	46.2 ± 12.4	47.3 ± 13.7
Female sex, n (%)	540 (64.8)	259 (40.5)	902 (57.6)	306 (36.1)
Symptom duration, y	13.0 ± 10.4	15.0 ± 11.1	15.0 ± 11.3	18.6 ± 12.5
HLA-B27 positive, n (%)	75 (17.2)	49 (17.4)	818 (58.6)	556 (73.1)
BMI, kg/m²	28.4 ± 5.7	27.4 ± 5.1	26.8 ± 5.5	25.6 ± 4.1
Obesity (BMI ≥ 30 kg/m²), n (%)	199 (32.9)	104 (25.4)	256 (24.1)	74 (12.8)
Current smoking, n (%)	120 (24.0)	57 (15.9)	268 (26.5)	126 (23.9)
CRP level, median (mg/L)	4.3	2.0	3.0	2.0
CRP level ≥ 5 mg/L, n (%)	333 (48.2)	167 (32.8)	479 (36.8)	187 (28.5)
ESR, mm/h	13.7 ± 12.4	10.4 ± 10.2	13.9 ± 14.2	10.8 ± 12.4
Disease activity and patient-reported outcomes				
MASES score, mean ± SD	2.8 ± 2.2	—	3.2 ± 2.5	—
TJC44	4.1 ± 6.1	1.2 ± 2.7	2.4 ± 5.6	0.3 ± 1.0
SJC44	1.4 ± 2.9	0.8 ± 2.1	0.7 ± 2.5	0.1 ± 0.8
Physician global (0-10)	3.1 ± 2.0	1.8 ± 1.8	3.1 ± 1.9	1.7 ± 1.7
Patient global (0-10)	4.4 ± 2.2	2.3 ± 2.1	4.7 ± 2.5	2.2 ± 2.0
ASDAS-CRP	—	—	2.6 ± 0.9	1.8 ± 0.9
BASDAI (0-10)	—	—	4.7 ± 2.1	2.8 ± 2.0
BASFI (0-10)	—	—	2.9 ± 2.3	1.5 ± 1.8
HAQ	0.4 ± 0.4	0.2 ± 0.3	—	—
Disease manifestations				
Peripheral arthritis, n (%)	730 (91.6)	590 (94.6)	746 (49.7)	352 (43.5)
Coxitis, n (%)	108 (13.7)	56 (9.2)	251 (16.9)	130 (16.1)
Dactylitis, n (%)	316 (40.3)	279 (46.0)	159 (10.8)	58 (7.3)
Uveitis, n (%)	32 (4.0)	26 (4.3)	237 (15.8)	159 (19.6)
Psoriatic skin manifestation, n (%)	633 (79.7)	540 (87.4)	139 (9.3)	77 (9.5)
Psoriatic nail manifestation, n (%)	316 (40.0)	300 (49.1)	43 (2.9)	23 (2.9)
Current treatment				
NSAID use, n (%)	464 (55.7)	220 (34.4)	859 (54.9)	298 (35.2)
csDMARD use, n (%)	318 (48.0)	202 (37.1)	206 (20.5)	83 (14.1)
bDMARD use, n (%)	442 (66.7)	402 (73.8)	905 (89.9)	540 (91.7)
tsDMARD use, n (%)	57 (8.6)	51 (9.4)	22 (2.2)	7 (1.2)

Values are mean ± SD or n (%). Disease-specific indices are shown where routinely assessed (axSpA: ASDAS-CRP, BASDAI, BASFI; PsA: HAQ).

ASDAS, ankylosing spondylitis disease activity score; axSpA, axial spondyloarthritis; BASDAI, Bath ankylosing spondylitis disease activity index; BASFI, Bath ankylosing spondylitis functional index; bDMARD, biologic disease-modifying antirheumatic drug; BMI, body mass index; CRP, C-reactive protein; csDMARD, conventional synthetic disease-modifying antirheumatic drug; ESR, erythrocyte sedimentation rate; HAQ, health assessment questionnaire; HLA-B27, human leukocyte antigen B27; MASES, Maastricht ankylosing spondylitis enthesitis score; NSAID, nonsteroidal anti-inflammatory drug; PsA, psoriatic arthritis; SJC44, swollen joint count (44 joints); TJC44, tender joint count (44 joints); tsDMARD, targeted synthetic disease-modifying antirheumatic drug.

Patients with enthesitis were more likely to receive NSAIDs and conventional synthetic csDMARDs. bDMARD use was more frequent in patients without enthesitis. tsDMARDs were more frequently prescribed in patients without enthesitis in PsA, whereas in axSpA they were slightly more common in patients with enthesitis. Among bDMARDs, TNF inhibitors were most used; among tsDMARDs, phosphodiesterase-4 inhibitors predominated in PsA and Janus kinase (JAK) inhibitors in axSpA, reflecting differences in approval status and time on the market.

### Pattern and anatomical distribution of enthesitis

Figure 2 illustrates MASES scores and the anatomical distribution of enthesitis among patients with a MASES score of > 0 at their index visit. Among patients with enthesitis and available information on axial involvement, the mean MASES score (± SD) was 2.8 ± 2.2 for PsA (n = 349), 3.0 ± 2.3 for axPsA (n = 382), and 3.2 ± 2.5 for axSpA (n = 1565), with scores ranging from 1 to 13 in both diseases. The most frequently clinically detected site was the posterior superior iliac spine, followed by the fifth lumbar spinous process. In PsA and axPsA, the Achilles tendon represented the third most frequently affected site. In contrast, in axSpA, other axial sites were more frequently involved, such as the first costochondral joint, the seventh costochondral joint, and the iliac crest. Notably, a substantial proportion of patients were affected bilaterally across several entheseal sites.

**Figure 2:**
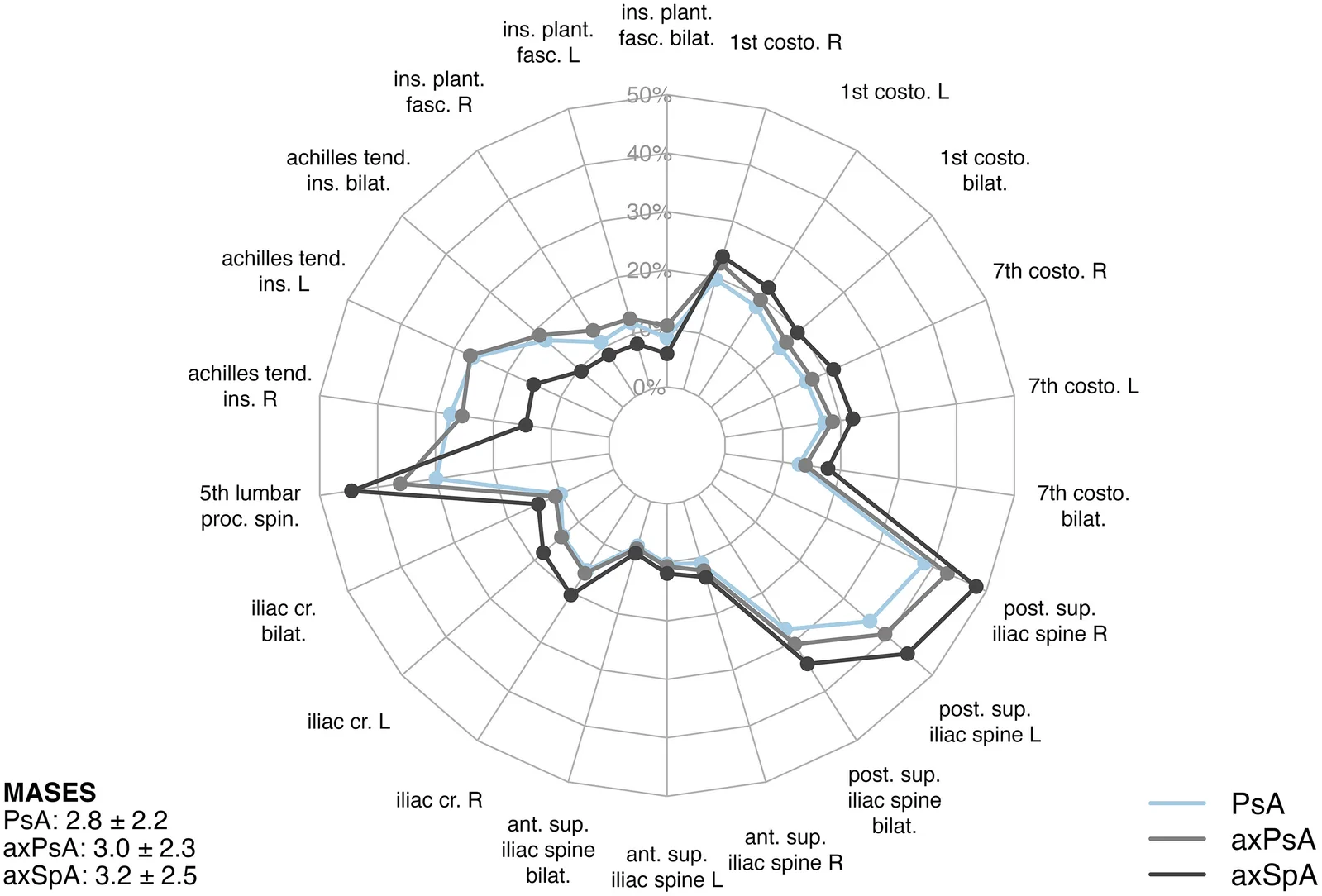
MASES score and anatomical distribution of enthesitis among patients with MASES scores > 0 at the index visit. 1st/7th costo., first/seventh costochondral joint; 5th lumbar proc. spin., fifth lumbar spinous process; Achilles tend. ins., Achilles tendon insertion; ant./post. sup. iliac spine, anterior/posterior superior iliac spine; axPsA, psoriatic arthritis with axial involvement; axSpA, axial spondyloarthritis; iliac cr., iliac crest; ins. plant. fasc., plantar fascia insertion; PsA, psoriatic arthritis; R/L/bilat., right/left/bilateral.

Figure 3 summarises factors associated with a higher enthesitis burden in patients with PsA (n = 843) and axSpA (n = 1349). In PsA, male sex (IRR, 0.42; 95% CI, 0.35-0.50), longer symptom duration (IRR, 0.99; 95% CI, 0.98-1.00), and treatment with bDMARDs (IRR, 0.68; 95% CI, 0.57-0.81) were associated with a lower enthesitis burden, whereas elevated CRP levels (IRR, 1.01; 95% CI, 1.00-1.02), overweight (IRR, 1.33; 95% CI, 1.08-1.63), obesity (IRR, 1.40; 95% CI, 1.13-1.72), and current smoking (IRR, 1.30; 95% CI, 1.00-1.69) were associated with a higher burden. In axSpA, male sex (IRR, 0.55; 95% CI, 0.48-0.63) and longer symptom duration (IRR, 0.99; 95% CI, 0.98-1.00) were associated with a lower burden, whereas elevated CRP levels (IRR, 1.01; 95% CI, 1.01-1.02), treatment with tsDMARDs (IRR, 1.81; 95% CI, 1.02-3.21), obesity (IRR, 1.47; 95% CI, 1.25-1.73), and current smoking (IRR, 1.24; 95% CI, 1.03-1.49) were associated with a higher burden.

**Figure 3:**
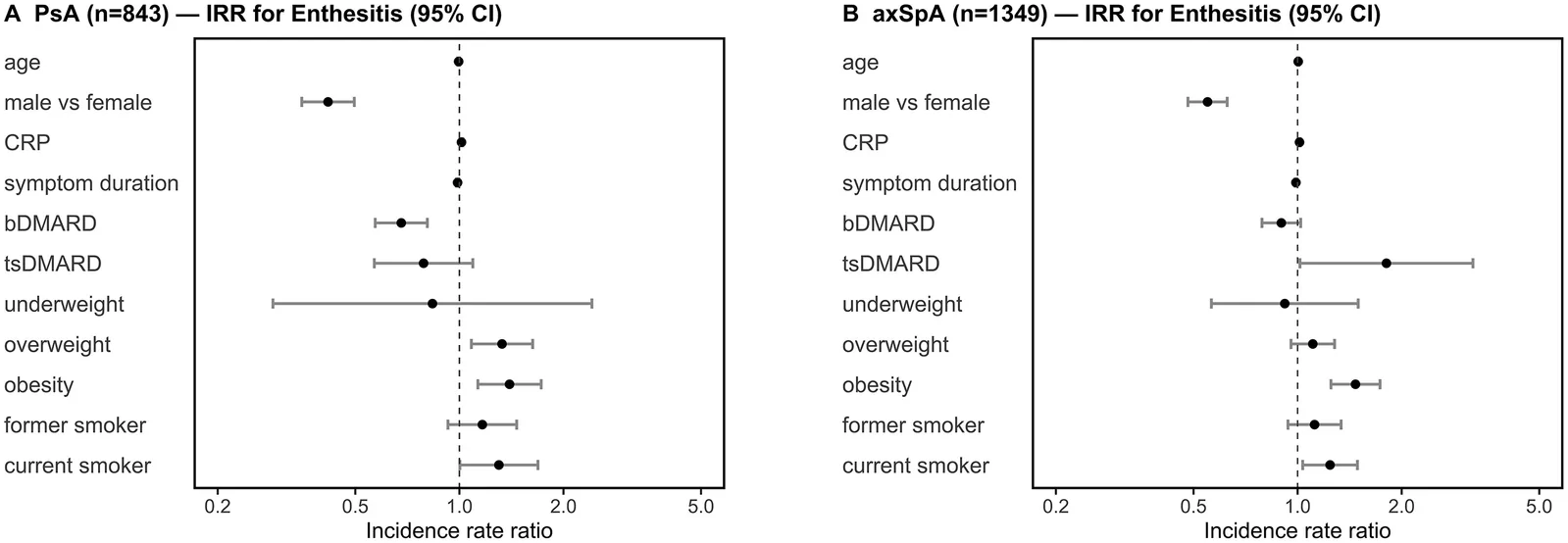
Factors associated with higher enthesitis burden (MASES scores, 0-13). axSpA, axial spondyloarthritis; bDMARD, biologic disease-modifying antirheumatic drug; BMI, body mass index; CRP, C-reactive protein; IRR, incidence rate ratio; MASES, Maastricht ankylosing spondylitis enthesitis score; PsA, psoriatic arthritis; ref., reference category; tsDMARDs, targeted synthetic disease-modifying antirheumatic drugs.

Table 2 summarises whether the associations of BMI score and smoking with enthesitis differ between PsA and axSpA and whether these associations vary by sex. Neither BMI nor smoking showed differential associations with enthesitis between the 2 diseases. Moreover, the magnitude of these associations was comparable in women and men.

**Table 2:** Interaction effects between disease and BMI or smoking (axSpA vs PsA) and 3-way interactions (disease × sex × predictor)

Contrast	IRR (95% CI)	*P* value
BMI (axSpA vs PsA)		
BMI overweight	0.90 (0.70-1.15)	.413
BMI obesity	1.11 (0.85-1.45)	.426
Smoking (axSpA vs PsA)		
Former smoker	1.01 (0.75-1.34)	.968
Current smoker	0.97 (0.70-1.33)	.839
Disease × BMI × sex		
Overweight	0.99 (0.59-1.67)	.981
Obesity	0.95 (0.54-1.66)	.855
Disease × smoking × sex		
Former smoker	1.46 (0.80-2.67)	.221
Current smoker	0.98 (0.50-1.92)	.958

axSpA, axial spondyloarthritis; BMI, body mass index; IRR, incidence rate ratio; PsA, psoriatic arthritis.

In a sensitivity analysis restricted to patients not receiving b/tsDMARDs at the index visit (PsA, n = 309; and axSpA, n = 484), the prevalence of enthesitis (MASES score > 0) was 55.7% in PsA and 57.0% in axSpA. Among patients with complete covariate data (PsA n = 181; axSpA n = 278), the direction and magnitude of associations were broadly consistent with the primary analysis: female sex (PsA: IRR, 0.30; 95% CI, 0.21-0.42; and axSpA: IRR, 0.49; 95% CI, 0.35-0.68) and CRP levels (IRR, 1.02 in both) remained significantly associated with enthesitis burden. However, the associations for BMI categories and smoking status did not reach statistical significance in this reduced sample, likely reflecting insufficient statistical power.

## DISCUSSION

This cross-sectional analysis of prospectively collected registry data from patients with PsA and axSpA in the SCQM registry indicates that, consistent with its predominantly axial inflammatory phenotype, axSpA is characterised by a higher enthesitis burden, predominantly at axial sites, compared with PsA. Patients with axPsA combined axial and peripheral involvement, bridging both phenotypes. The occurrence of enthesitis followed comparable patterns in PsA and axSpA, with consistent associations for systemic inflammation (CRP levels), sex, symptom duration, BMI scores, and smoking status. These shared associations suggest that key correlates of enthesitis are not disease-specific but reflect common mechanisms across the spondyloarthritis spectrum. Notably, modifiable factors including obesity and active smoking showed broadly similar associations irrespective of diagnosis or sex, suggesting their potential relevance as therapeutic and preventive targets.

The prevalence of enthesitis in our analysis was slightly higher in PsA (56.6%) and axSpA (64.9%) than in comparable studies from the literature, which report prevalence estimates ranging from 25% to 53% in PsA to 21% to 56% in axSpA [10,[13], [14], [15]]. This difference may be explained by our inclusion criteria, as only patients with a documented MASES score assessment at the index visit were considered. It is therefore plausible that the MASES score assessment was performed less frequently in the absence of enthesitis symptoms, which may have led to a slight overestimation of the prevalence in our study. Our observation that patients with axSpA exhibit greater enthesitis burden than those with PsA, based on average MASES scores (PsA, 2.8 ± 2.2; and axSpA, 3.2 ± 2.5), is only rarely reflected in existing studies. Studies assessing enthesitis burden demonstrate a strong dependence on the index applied. Investigations using the SPARCC index, which primarily evaluates peripheral entheses, generally report a greater burden in PsA, whereas studies using the MASES score, which emphasises axial sites, are scarce [10,14]. Nevertheless, several reports suggest that mean MASES in PsA typically ranges between 1.4 and 2.9, whereas corresponding estimates in axSpA cohorts tend to fall between 3 and 4 [7,13,16]. Published data describing enthesitis in axPsA remains limited. Overall, our findings suggest that patients with axPsA appeared to show an anatomical distribution of enthesitis that shared features of both PsA and axSpA [4,5].

Across PsA, axPsA, and axSpA, the posterior superior iliac spine emerges as the most frequently affected site of enthesitis, followed by the spinous process of the fifth lumbar vertebra. In addition, the Achilles tendon in PsA and the first and seventh costochondral joints in axSpA were also commonly involved. These localisations are in good agreement with the current literature [13,14,17,18]. Importantly, our findings are consistent with previous reports demonstrating that axSpA is characterised by a predominantly axial enthesitis burden, whereas PsA is more commonly associated with peripheral enthesitis [10,17,19]. A recent study demonstrates that patients with PsA, irrespective of enthesitis location, exhibit higher disease activity than those without enthesitis. These findings highlighted the crucial role of a complete enthesitis assessment in clinical practice [20].

So far, only a few studies have addressed the association between enthesitis and sex. Consistent with our findings, the studies report a significantly higher prevalence of enthesitis in women compared with men [14,21,22]. Possible explanations include a higher sensitivity to pain and a greater likelihood of pain reporting among women [23]. Moreover, conditions such as fibromyalgia and widespread nonarthritic pain, often linked to central sensitisation, are reported to occur more frequently in women [24,25]. Due to the absence of data on fibromyalgia, its potential influence on enthesitis could not be assessed. Studies indicate that the coexistence of enthesitis and fibromyalgia is associated with higher clinical enthesitis scores. However, this association does not necessarily correspond to increased inflammatory findings on ultrasound [26].

Patients with enthesitis in both disease groups have significantly higher BMI scores compared with those without enthesitis. This observation aligns with previous reports indicating that obesity is associated with musculoskeletal inflammation, although some studies have found no significant association between BMI and the presence of enthesitis [7,[27], [28], [29]]. Several mechanisms may underlie this association. Increased body weight is associated with greater mechanical load at entheseal sites, which may promote microtrauma and contribute to local inflammation [30,31]. In addition, adipose tissue is metabolically active and secretes proinflammatory mediators such as adipokines, which may further enhance systemic and local inflammation [32]. Moreover, a recent study demonstrates that obesity may also reduce the efficacy of pharmacologic treatments [[33], [34], [35]]. Taken together, these findings suggest that a higher BMI score may be associated not only with a higher likelihood of enthesitis but also with its persistence.

Consistent with previous studies, patients with PsA and axSpA who present with enthesitis in our cohort exhibit significantly higher disease activity, poorer PROMs, and greater symptom burden compared with those without enthesitis [20,36]. This increased disease burden is also reflected in treatment patterns. Patients with enthesitis were more frequently treated with NSAIDs. Interpretation of bDMARD and tsDMARD use is complicated by 2 opposing biases. Confounding by indication, since patients with greater disease burden and prior treatment failure are more likely to receive these medications, and treatment-related misclassification, as effective therapy may resolve enthesitis and thereby reclassify patients into the group without enthesitis.

Our analysis highlighted both shared and disease-specific factors associated with enthesitis in PsA and axSpA. In both conditions, male sex and longer symptom duration were associated with a lower enthesitis burden. Conversely, elevated CRP levels emerged as a common correlate with higher enthesitis load in both diseases. Modifiable factors such as obesity and smoking were consistently associated with a higher burden of enthesitis across PsA and axSpA. However, no differences in the associations of BMI scores and smoking status with enthesitis were observed between PsA and axSpA, and the magnitude of these associations did not differ by sex. Studies investigating the factors associated with enthesitis are scarce. However, the limited available evidence suggests similar associations, especially concerning elevated CRP levels, as an indicator of ongoing systemic inflammation, alongside female sex and higher BMI, as previously discussed [37]. Studies show conflicting results regarding symptom duration, but its effect on enthesitis has only been investigated to a limited extent so far [38,39]. However, a potential bias needs to be considered. With longer symptom duration, therapy is likely to be more optimised, which may have already led to the resolution of enthesitis through targeted treatment, resulting in patients being classified into the group without enthesitis. Consequently, the group without enthesitis may also comprise patients with successfully treated enthesitis. Like symptom duration, the impact of smoking on enthesitis remains uncertain according to the available studies. Most studies do not directly investigate the effect of smoking on enthesitis, but rather the influence of smoking on inflammation and disease activity. Whereas data on axSpA suggest a link between smoking, inflammation, and disease activity, the effect of smoking on PsA remains inconclusive [[40], [41], [42], [43]]. Treatment-related associations need to be interpreted with caution. In PsA, bDMARD use was associated with a lower enthesitis burden, which may partly reflect the efficacy of biologic therapies in reducing entheseal inflammation, although reverse causality (ie, effective treatment resolving enthesitis) cannot be excluded in this cross-sectional design [44]. In axSpA, however, tsDMARD use was associated with a higher enthesitis burden (IRR, 1.81; 95% CI, 1.02-3.21), which almost certainly reflects confounding by indication: in the SCQM registry, JAK inhibitors predominate among tsDMARDs in axSpA and are typically reserved for patients with bDMARD-refractory or more severe disease, who would be expected to carry a higher residual disease burden irrespective of treatment. This interpretation is supported by randomised controlled trial evidence showing inconsistent enthesitis-specific efficacy of JAK inhibitors in axSpA: whereas upadacitinib demonstrated nominal MASES score improvement in SELECT-AXIS 1 and 2, tofacitinib failed to achieve a significant reduction in MASES scores in its phase 3 ankylosing spondylitis trial [45,46]. By contrast, IL-17 inhibitors such as secukinumab and ixekizumab have demonstrated substantially higher rates of MASES score resolution compared with placebo in axSpA [47]. The predominance of JAK inhibitors as tsDMARDs in the SCQM registry of patients with axSpA—often initiated after TNFi and/or IL-17i failure—thus biases towards a treatment-refractory, high-enthesitis phenotype and should not be interpreted as a causal drug effect [48].

A major strength of this cross-sectional study is the large sample size of patients diagnosed with PsA and axSpA drawn from the SCQM registry. This well-established and high-quality database with detailed patient-level documentation enabled a direct comparison of PsA and axSpA in relation to enthesitis using validated scores. Finally, the study provided the largest real-world comparative analysis of enthesitis burden and determinants across PsA and axSpA to date, using a geographically defined nationwide registry with standardised data collection.

A limitation of this study is that the data quality of the registry depends on the accuracy and completeness of documentation by participating physicians. Accordingly, regression analyses were restricted to patients with complete data for all prespecified covariates (complete case analysis); the extent to which excluded patients differed systematically from those included—for example, by having less well-documented disease activity—could not be formally assessed and may represent an additional source of bias. Consequently, not all variables were available for every patient, which inevitably affected the overall data quality. Another limitation is that axial involvement in PsA was defined according to the treating physician’s assessment rather than a standardised definition combining clinical and imaging findings, which may have resulted in some degree of misclassification. Furthermore, our study examined patients only at a single time point, the index visit, which could represent either an initial consultation or a routine follow-up. Therefore, disease progression was not captured, and patients classified as not having enthesitis may have developed it later, whereas those with enthesitis at baseline may have experienced resolution outside the observation window. Another noteworthy limitation is the risk of selection bias. A sensitivity analysis excluding patients on b/tsDMARDs showed directionally consistent results but remained inconclusive for BMI scores and smoking, likely reflecting insufficient power in the reduced sample. Furthermore, no data on fibromyalgia and central sensitisation were considered, although these factors may confound enthesitis [49].

These insights may support more tailored assessment and management strategies for patients with PsA and axSpA, particularly by addressing modifiable risk factors such as obesity and smoking. Further studies, especially longitudinal analyses, are warranted to validate these findings, clarify causal relationships, and inform strategies for more personalised management of enthesitis in PsA and axSpA.

Taken together, this study provided new insights into the burden, distribution, and determinants of enthesitis across PsA and axSpA. Our findings emphasised the greater axial enthesitis burden in axSpA compared with PsA; suggested that axPsA shares anatomical enthesitis features of both PsA and axSpA; and highlighted female sex, systemic inflammation, and modifiable risk factors, including BMI scores and smoking, as common correlates of enthesitis burden across diagnoses.

## Editor disclosure

The peer review process did not involve Editorial Board Member Oliver Distler, and the editorial decision-making was led by editors who were not involved in the creation of this manuscript.

## CRediT authorship contribution statement

**Felix Doll:** Writing – review & editing, Writing – original draft, Visualization, Methodology, Investigation, Formal analysis, Conceptualization. **Adrian Ciurea:** Writing – review & editing, Methodology, Investigation. **Sabine Adler:** Writing – review & editing, Investigation. **Laure Brulhart:** Writing – review & editing, Investigation. **Frauke Förger:** Writing – review & editing, Investigation. **Thomas Hügle:** Writing – review & editing, Investigation. **Diego Kyburz:** Writing – review & editing, Investigation. **Burkhard Möller:** Writing – review & editing, Investigation. **Michael J. Nissen:** Writing – review & editing, Investigation. **Giorgio Tamborrini:** Writing – review & editing, Investigation. **Oliver Distler:** Writing – review & editing, Investigation. **Julia-Tatjana Maul:** Writing – review & editing, Investigation. **Raphael Micheroli:** Writing – review & editing, Supervision, Methodology, Investigation, Conceptualization.

## Competing interests

SA received consulting fees, speaker fees, and/or support for attending meetings from AbbVie, Eli Lilly, Janssen, GSK, Novartis, Pfizer, and Roche. FF received research grants from Novartis, GSK and UCB, consulting fees from UCB, Roche and Otsuka, and speaker fees from UCB, Mepha and MSD. TH received grants, consulting fees, and/or speaker honoraria from AbbVie, GSK, Johnson & Johnson, AstraZeneca, Novartis, and Mpriq, and is a shareholder of Atreon S.A. DK received a research grant from AbbVie, honoraria for presentations from AbbVie and Eli Lilly, support for attending meetings from Janssen and Eli Lilly, and payments for participation on advisory boards from AbbVie, Eli Lilly, Janssen, Novartis, Pfizer and Roche. MJN received consulting and/or speaker fees from AbbVie, Eli Lilly, Janssen, Novartis, and Pfizer, as well as a research grant from Novartis. JTM has served as an advisor and/or received speaker fees from AbbVie, Almirall, Amgen, BMS, Celgene, Eli Lilly, LEO Pharma, Incyte, Janssen-Cilag, MSD, Novartis, Pfizer, Pierre Fabre, Roche, Sanofi and UCB. OD has or has had consultancy relationships with, has received research funding from, and/or has served as a speaker for the following companies in the last three calendar years: 4P-Pharma, AbbVie, Acepodia, Aera, AnaMar, Anaveon, argenx, AstraZeneca, Avalyn, Boehringer Ingelheim, BMS, Calluna, Cantargia, CSL Behring, EMD Serono, Galderma, Galapagos, Gossamer, Hemetron, Innovaderm, Kali, Lilly, Mediar, MSD Merck, Nkarta, Novartis, Oorja Bio, Orion, Pliant, Prometheus, Quell, Scleroderma Research Foundation, Skyhawk, Tandem, Topadur, UCB and Umlaut.bio. OD is also a co-founder of CITUS AG. RM received speaker fees from AbbVie, Johnson & Johnson, UCB, and Eli Lilly. FD, AC, LB, BM, and GT have no competing interests.
